# Environmental and Biotic Correlates to Lionfish Invasion Success in Bahamian Coral Reefs

**DOI:** 10.1371/journal.pone.0106229

**Published:** 2014-09-03

**Authors:** Andrea Anton, Michael S. Simpson, Ivana Vu

**Affiliations:** 1 Curriculum for the Environment and Ecology, University of North Carolina, Chapel Hill, North Carolina, United States of America; 2 Department of Biology, University of North Carolina, Chapel Hill, North Carolina, United States of America; Institute of Marine Research, Norway

## Abstract

Lionfish (*Pterois volitans*), venomous predators from the Indo-Pacific, are recent invaders of the Caribbean Basin and southeastern coast of North America. Quantification of invasive lionfish abundances, along with potentially important physical and biological environmental characteristics, permitted inferences about the invasion process of reefs on the island of San Salvador in the Bahamas. Environmental wave-exposure had a large influence on lionfish abundance, which was more than 20 and 120 times greater for density and biomass respectively at sheltered sites as compared with wave-exposed environments. Our measurements of topographic complexity of the reefs revealed that lionfish abundance was not driven by habitat rugosity. Lionfish abundance was not negatively affected by the abundance of large native predators (or large native groupers) and was also unrelated to the abundance of medium prey fishes (total length of 5–10 cm). These relationships suggest that (1) higher-energy environments may impose intrinsic resistance against lionfish invasion, (2) habitat complexity may not facilitate the lionfish invasion process, (3) predation or competition by native fishes may not provide biotic resistance against lionfish invasion, and (4) abundant prey fish might not facilitate lionfish invasion success. The relatively low biomass of large grouper on this island could explain our failure to detect suppression of lionfish abundance and we encourage continuing the preservation and restoration of potential lionfish predators in the Caribbean. In addition, energetic environments might exert direct or indirect resistance to the lionfish proliferation, providing native fish populations with essential refuges.

## Introduction

Establishment of non-native species in new biogeographic regions can have serious consequences on biodiversity [1] and is now recognized as one the world's most critical conservation challenges [2]. Both physical and biological characteristics of the new environment affect the fate and success of exotic species [3]–[5]. Clearly, the physical environment must be physiologically tolerable: harsh environments such as deserts have been shown to be the least invaded worldwide [6], perhaps because the suite of non-native species pre-adapted to those extreme conditions is limited. Alternatively, when environmental conditions are tolerable, biotic resistance may inhibit local invasion success [7]. Biotic resistance stems from community diversity [8] or from the effects of strong local enemies (e.g. predators, competitors, or pathogens), affecting the fate of the exotic species in the new range. For instance, the native blue crab (*Callinectes sapidus*) provides biotic resistance against invasion by green crabs (*Carcinus maenas*) through direct predation in eastern North America [9]. Similarly, communities are more susceptible to invasion if they provide essential resources [10] or if the exotic species outcompetes native species in resource acquisition. For instance, invasive Argentine ants (*Linepithema humile*) outcompete native ants for food sources, depressing native ant abundance in northern California [11].

Invasive lionfish (*Pterois volitans*), a native species from the Indo-Pacific, was first detected in Florida in 1985 [12] and spread rapidly throughout the tropical Caribbean, subtropical southeast Atlantic coast [13] and has been recently spotted in the Mediterranean Sea [14]. This particular invasion is now ranked as one of the top-ten most serious emerging environmental issues in the world [15]. Densities of lionfish in their new biogeographic region are up to 15 times those in their native environment [16]. On reefs in the Bahamas, lionfish consume small fish and are thereby capable of reducing native fish abundance [17], biomass [18], and richness [19]. These findings are consistent with a meta-analysis that reveals that some novel predators can exert impacts on prey populations roughly double that of native predators [20]. Possible explanations for the successful lionfish invasion of the Atlantic include its diet breadth, comprising dozens of species of native fishes [see Table S1 for a list of fish species that are lionfish current prey in the Atlantic and Caribbean], naiveté of prey towards exotic lionfish [21], [22], and the possibility of a geographic escape from control by natural enemies [22], [23] (although see [24], [25]). Threats posed by invading lionfish are particularly serious because of the high ecological and economic values of coral reefs in the Caribbean [26]. Similarly, lionfish are a threat to reefs in southeastern North America [27], [28] which are habitat for valuable reef fishes of the snapper-grouper complex already seriously stressed by overfishing [29].

Here we utilize the invasion of lionfish in reefs of San Salvador, Bahamas, first documented in 2005 [13] to quantify potentially important physical and biological environmental characteristics to determine which factors contribute to the success of the lionfish invasion. We operationally define invasion success by lionfish abundance, either density or biomass. By assessing the effect of wave exposure on lionfish abundance, we test how physical energy relates to lionfish invasion success on coral reefs. By evaluating how the rugosity of the reefs affects lionfish abundance, we measure the role of structural complexity on the invasion process. By exploring how lionfish abundance relates to abundances of large native groupers and other predatory fishes, we infer whether biotic resistance to invasion may be provided by natural predators or competitors. Finally, by relating lionfish abundance to abundance of small and medium fishes, we infer whether prey availability may be facilitating lionfish invasion success.

## Methods

### Ethics statement

No protected or endangered species were involved in this field study. Surveys were performed through visual census and no vertebrates were handled or collected. Approvals by the Department of Marine Resources of The Bahamas were obtained to perform the surveys.

### Field Sampling

**Figure 1:**
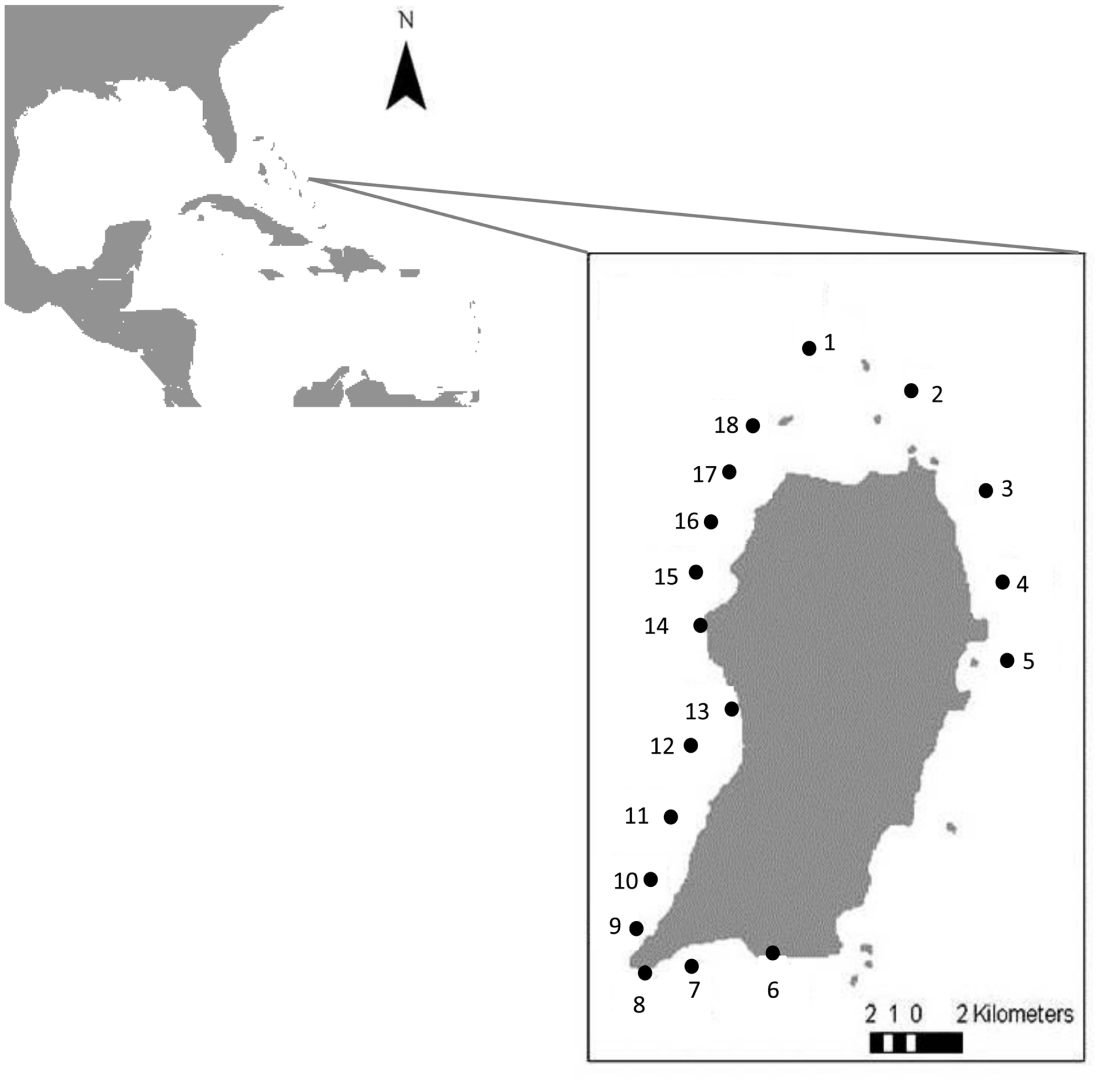
Map of study sites in the island of San Salvador (The Bahamas). Circles indicate the study sites. Numbers indicate study sites as follows: (1) White Island, (2) Catto Cay, (3) Light House, (4) Baptism, (5) Crab Cay, (6) La Crevasse, (7) Danger Point, (8) Double Caves, (9) Grotto, (10) Great Cut, (11) Red House, (12) Gardness, (13) Sangrila, (14) Runway, (15) Club Med, (16) Yellow House, (17) Rocky Point, and (18) Green Cay. This map was generated using a publicly available shapefile from the World Resources Institute (World Resources Institute website. Available: http://www.wri.org. Accessed 2014 August 7).

We conducted field surveys at 18 sites around the island of San Salvador, Bahamas in July-August 2009, in coral reef habitat at depths between 13–17 m (Fig. 1). Lionfish were detected in San Salvador in 2005 [13]. Sites were separated by more than 1.5 km. Buoys, which are used to moor boats over the reefs, were avoided when selecting sites to minimize possible influences of spearfishermen on lionfish. Three replicate, haphazardly placed 50 m long transects, separated by approximately 20 m gaps, were deployed to perform surveys of benthic habitat cover and fish abundances at each site. Transects were oriented parallel to shore and surveys were conducted between 9:00–16:00 h. On each transect, divers working together but on different sections along the transect followed a sequence of sampling protocols (Fig. S1). Fish surveys were performed using standard underwater visual belt transect methods [30], which were conducted by two divers for safety reasons. One diver quantified lionfish and large (>30 cm in total length, TL) native predatory fish abundances by species (listed in Table S2), and estimated TL of each individual within 500 m^2^ (50×10 m; large quadrat) area along the transect (Fig. S1). Care was taken to examine cryptic habitats by thoroughly inspecting reef crevices and overhangs, to avoid underestimating lionfish densities [31]. Simultaneously another diver quantified potentially suitable prey fishes of two different sizes: Fishes of less than 5 cm total length (TL), termed small fishes, were quantified in 30 m^2^ (15×2 m; small quadrat) area and prey fishes of 5–10 cm TL, termed medium fishes, were counted in 120 m^2^ (30×4 m; medium quadrat) area (Fig. S1). At each transect, the large quadrat contained the medium and small quadrats and the medium quadrat did not overlap with the small quadrat (Fig. S1). To reduce the effect of one diver on the observations of a second diver, the two divers that perfomed the fish surveys advanced simultaneously along the transect line, with the diver examining the large quadrat performing 1-way ziz zag swims centered on the transect, while the second diver progressed in a straight forward motion along the transect. Small quadrats were surveyed after the large and medium quadrats to minimize any influence on large fish. Fishes were recorded by species except nocturnal (such as Apogonidae) and highly cryptic (such as Blenniidae and Gobiidae) fish species, that were not quantified, following other comparable previous studies [32]. Only small and medium fish species identified in the literature as lionfish prey were included in the statistical analysis (Table S1 and Table S2).

Fish counts from the three same-size quadrats surveyed per site were pulled together to calculate fish counts per site per unit of area surveyed to avoid pseudoreplication and spatial autocorrelation (transects in the same site are more similar that transects from other sites). Small and medium fish counts were assessed per unit of area (small fish of 0–5 cm TL in 90 m^−2^ area and medium fish of 5–10 cm TL in 360 m^−2^ area) and then extrapolated to individual 1500 m^−2^ to aid comparison with the fish counts in the larger transect. Fish densities for each species were transformed to biomass by using the allometric length-weight conversion formula *W = aTL^b^*, where *W* is the weight of each individual fish in grams, *TL* is the total length recorded for each fish in cm and the parameters *a* and *b* are species-specific constants. The parameters *a* and *b* for each species were obtained from FishBase ([33]; Table S2). Lionfish lengths (cm) were converted to biomass (g) using empirically fitted, allometric scaling parameters (a = 0.00492 and b = 3.31016) obtained from the weight and length of 137 lionfish from Abaco Island, Bahamas [22].

### Environmental predictors: Wave exposure and habitat complexity

**Figure 2:**
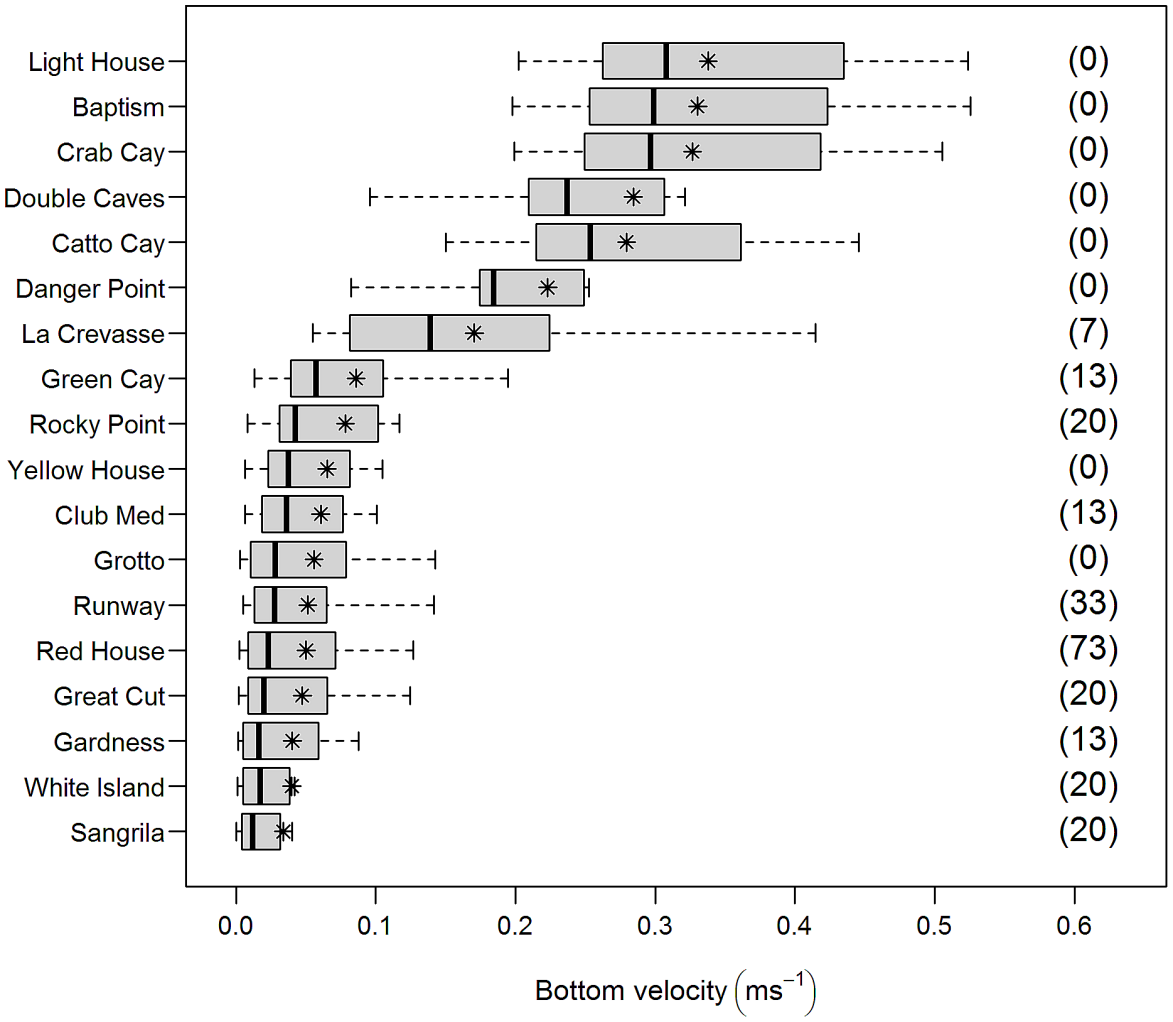
Boxplots of bottom velocity averaged over summer months as a function of site. Mean bottom velocity (m s^−1^) at 18 sites in San Salvador Island (Bahamas) for the summer months (May, June, July, and August). Sites were ordered, listed with increasing mean bottom velocities. The wave parameters used to calculate bottom velocity were collected hourly by oceanic buoys. Lionfish mean density (individuals ha^−1^) per site was indicated in parenthesis.

We estimated the average bottom velocity (i.e. velocity of the water near the sea floor) at each site as a metric of the degree of wave exposure to demersal and semi-demersal fish species. Land masses can modify the wave energy near the bottom and wave exposure was calculated as follows. First, we determined vectors of the oceanic waves that could strike each site (all directions from which the waves could reach a site) in San Salvador using geographic maps. Bottom velocity depends on wave direction, dominant wave period, wave height and depth [34]. Depth was measured *in situ* on each study site using dive computers. Wave direction, dominant wave period, and wave height were obtained from data available online (National Data Buoy Center website. Available: http://www.ndbc.noaa.gov/. Accessed 2014 August 7) from two permanent moored buoys (41047-NE Bahamas and 41046-East Bahamas) owned and maintained by the National Oceanic and Atmospheric Administration. We assumed that the same waves that were reaching these buoys also reached our study sites. The historical public record of wave data from buoys is intermittent but included data from May to December 2009, January to July 2010 and January to December 2011 from the NE Bahamas buoy and data from August to December 2010 from East Bahamas buoy. Buoys collect data hourly from which we estimated bottom velocity [34] hourly for each site for all waves that directly reached that site: otherwise bottom velocity was recorded as zero. We then computed monthly average bottom velocities for every study site over all the time periods (above) for which these buoys recorded wave data. We used estimated site means of bottom velocities from May through August (“summer” months) to construct box plots of the hourly bottom velocity for each month, allowing visual comparison of wave exposure between sites (Fig. 2). This time period includes the field sampling months of July and August plus the two preceding months, which could also have strong influences on biotic patterns.

Topographic complexity (e.g., the rugosity of the reef) was measured on each transect (3 times per site) by carefully laying a 30 m steel chain (2 cm long links) to the reef surface. The chain was deployed following the length of the measuring tape used in the fish surveys. A rugosity index (C) was calculated per site as *C = 1−d/l*, where *d* is the horizontal distance covered by the chain when following the contour of the reef and *l* is the length of the chain when fully extended (30 m; [35], [36]).

In addition, we quantified benthic habitat cover along 30 m of the transect line placed on the bottom at each site to investigate the effects of environmental predictors of benthic habitat. We classified benthic habitat type as coral cover (including a subsection of important reef-building corals-the *Orbicella* habitat, which comprised *Montastraea annularis*, *M. flaveolata* and *M. franksi* species), macroalgae cover, turf algae cover, sponge cover, and gorgonian cover. Other benthic habitat types (sand, cyanobacteria and crustose coralline algae) were also identified but not used in this study because they provide no emergent habitat structure for fish. We identified habitat category at 50 cm intervals directly below marked points on the transect line. Benthic cover measurements were pooled by transect and then averaged across transects to produce site means for each habitat type.

### Statistical analyses

A subset of large, native predatory groupers (so forth collectively termed “large grouper”), consisted of Nassau grouper (*Epinephelus striatus*) and tiger grouper (*Mycteroperca tigris*), were selected to aid comparison of findings with previous studies ([24]; Table S2).

First, to corroborate the independence between model predictors, we tested for correlation among our abiotic (wave exposure and structural complexity) and biotic (small fish-, medium fish-, large grouper-, large predatory fish- density or biomass) independent variables by creating a Pearson's product-moment correlation coefficients table (Table S3). The correlation matrix indicated no correlation among preditors with the exception of 1) wave exposure and small fish density and biomas and 2) large predatory fish density and large grouper density (Table S3). To avoid data missinterpredation arising from models with correlated predictors, we dropped small fish density and biomass from the analyses while large predatory fish abundance and large grouper abundance were not included together as predictors in the same statistical analyses.

We employed generalized linear models to determine the effect of biotic (medium fish abundance, large predator abundance, and grouper abundance) and abiotic factors (wave exposure and rugosity) on lionfish abundance (density and biomass). Four independent generalized linear models were run to test the effect of the environmental and fish variables on lionfish abundance: 2 models had lionfish density as dependent variable and the other 2 models had lionfish biomass as dependent variable (Table 1). Fish independent variables were either medium fish abundance and large predatory fish abundance or medium fish abundance and large grouper abundance (Table 1). We ran separate analysis with grouper abundance (instead of predator abundance) as a predictor because 1) grouper abundance was contained within large predator abundance, 2) large predatory fish density and large grouper density were significantly correlated (Table S3) and 3) to allow comparisons with previous studies [24]. Models with were fitted by negative binomial distributions on zero-inflated models (ZINB) because lionfish data distributions were heavily sckewed towards zero, containing more zeros than expected based on a negative binomial distribution [37]. A ZINB model is a mixture model consisting of two parts: A binomial model (zero-inflation model) that accounts for the excess zeros and a count model that includes the counts and the expected zeros modeled with a negative binomial distribution [37]. Both parts of the mixture model can include independent variables. We did not include predictors in the zero-inflated part of the model because we do not suspect that the probability of false zeros was a function of any of our predictors [37]. To validate the models we plotted the model residuals against the fitted values and no patterns were detected in any of the four models. No interaction terms were included in the models to avoid overfitting. The range of large predator and large grouper biomass was 100 and 9 times larger than lionfish biomass respectively (Table 2) and these predictors were standardized (centered and scaled by sustracting the mean and dividing by the standard deviation) to improve model convergence.

**Table 1:** Statistical zero-inflated negative binomial models for the effects of environment (wave exposure and structural complexity), and fish (small and medium fishes, and large predatory fishes) abundance (density and biomass) on lionfish abundance (density and biomass).

Dependent variable	Independent variable	Coefficient Estimate	SE*	z-value	p-value
Lionfish density	Intercept	1.445	1.598	0.904	0.365
(individuals 1500 m^−2^)	Medium fish density	−0.007	0.003	−1.958	0.05
	Large predator density	0.076	0.031	2.382	**0.017**
	Rugosity	1.406	4.406	0.319	0.75
	Wave exposure	−14.847	5.4	−2.749	**0.006**
Lionfish density	Intercept	1.127	1.7163	0.657	0.511
(individuals 1500 m^−2^)	Medium fish density	−0.002	0.004	−0.45	0.653
	Large grouper density	0.197	0.136	1.444	0.149
	Rugosity	1.603	5.228	0.307	0.759
	Wave exposure	−15.636	6.531	−2.394	**0.016**
Lionfish biomass	Intercept	5.983	2.209	2.368	0.008
(g 1500 m^−2^)	Medium fish biomass	−9.586	<−0.001	−1.098	0.272
	Large predator biomass†	−0.276	0.301	−0.891	0.373
	Rugosity	10.41	6.361	1.636	0.102
	Wave exposure	−42.56	8.95	−4.755	**<0.001**
Lionfish biomass	Intercept	5.681	2.437	2.331	0.019
(g 1500 m^−2^)	Medium fish biomass	−0.001	<−0.001	−1.763	0.078
	Large grouper biomass†	−0.235	0.368	−0.639	0.523
	Rugosity	11.79	7.725	1.527	0.126
	Wave exposure	0.059	11.32	−3.872	**<0.001**

The four models had 7 degrees of freedom.

† This variable was centered and scaled

Bolded values denote significant differences at p<0.05

**Table 2:** Conversion table with the density and biomass of lionfish, medium fish, large predatory fish and large grouper.

	Density, individuals 1500 m^−2^	Biomass, g 1500 m^−2^
Lionfish	1.94±2.7 (0–11)	409±674 (0–2591)
Medium fish	81±48 (8–162)	646±406 (102–1434)
Large predatory fish	5.9±6.7 (0–22)	42471±80070 (0–292213)
Large grouper	1.55±1.61 (0–6)	3580±3912 (0–13221)

	Density, individuals ha^−1^	Biomass, g 100 m^−2^
Lionfish	13±18 (0–73)	27.3±44.9 (0–173)
Medium fish	540±320 (53–1080)	4306±2706 (680–9560)
Large predatory fish	39±45 (0–147)	2831±5338 (0–19481)
Large grouper	10±11 (0–40)	238±261 (0–881)

Values are presented as mean ± standard deviation (minimum value-maximum value).

To elucidate potential indirect bottom-up effects of wave exposure or reef rugosity on lionfish abundance, we performed correlations among each habitat type (the % cover of corals, the *Orbicella* habitat, macroalgae, turf algae, sponges and gorgonias) and the abiotic variables (wave exposure and reef rugosity) by creating a Pearson's product-moment correlation coefficients table (Table 3).

**Table 3:** Table of the Pearson's product-moment correlation coefficients between benthic habitat type (coral cover, macroalgae cover, turf algae cover, *Orbicella* habitat cover, sponge cover and gorgonian cover) and abiotic predictors (wave exposure and rugosity).

Variable	Rugosity (C)	Wave exposure (m s^−1^)
Coral cover (%) [10.5±5.1]	0.129	0.044
Macroalgae cover (%) [61.1±21.1]	−0.116	*−0.83
Turf cover (%) [10.9±10.4]	0.233	*0.874
*Orbicella* habitat cover (%) [3.1±2.8]	0.096	0.353
Sponge cover (%) [1.4±1.7]	−0.212	−0.211
Gorgonian cover (%) [1.2±1.2]	0.309	0.040

The asterisk (*) indicates significant differences at p-values <0.05.

Finally, a clear separation existed between sites with relatively low and high wave exposure (7 versus 11 sites respectively; see Fig. 2): To corroborate our results on the effect of wave exposure on lionfish abundance (density and biomass) and reef rugosity, we performed three additional statistical analyses. First, we ran two independent zero-inflated generalized linear models to test for differences in lionfish density or biomass across sites with low and high wave exposure. Second, we used a two sample one-tailed t-test to assess differences in reef rugosity between low and high wave exposure sites. All statistical analyses were performed with R version 3.1.0 (R project for Statistical Computing website. Available: http://www.r-project.org. Accessed 2014 August 7.) in RStudio (RStdio website. Available: https://www.rstudio.com/. Accessed 2014 August 7.) with packages MASS [38] and pscl [39].

## Results

To facilitate the comparison of fish abundances with previous studies, we built a table (Table 2) that includes the fish counts and their calculated biomass per site (individuals 1500 m^−2^ and grams 1500 m^−2^ respectively) and also a conversion of the same variables into the common units used in the literature to report fish abundance (density as individuals ha^−1^ and biomass as g 100 m^−2^).

Summer-time estimated near-bottom velocities for the 4 years of buoy wave data (mean per site) ranged from 0.033 to 0.337 with a mean of 0.14 (±0.12) m s^−1^ (Fig. 2) and rugosity index (C) ranged from 0.021 to 0.57 with a mean of 0.37 (±0.13). Lionfish density in our study ranged from 0 (in eight sites) to 73 with a mean (±SD) of 13 (±18) individuals ha^−1^ (Table 2 and Fig. 2) and lionfish biomass ranged from 0 to 173 with a mean of 27 (±45) g 100 m^−2^ (Table 2). Medium fish, large predatory fish, and large grouper mean densities were 540 (±320), 39 (±45), and 10 (±11) individuals ha^−1^ respectively (Table 2).

**Figure 3:**
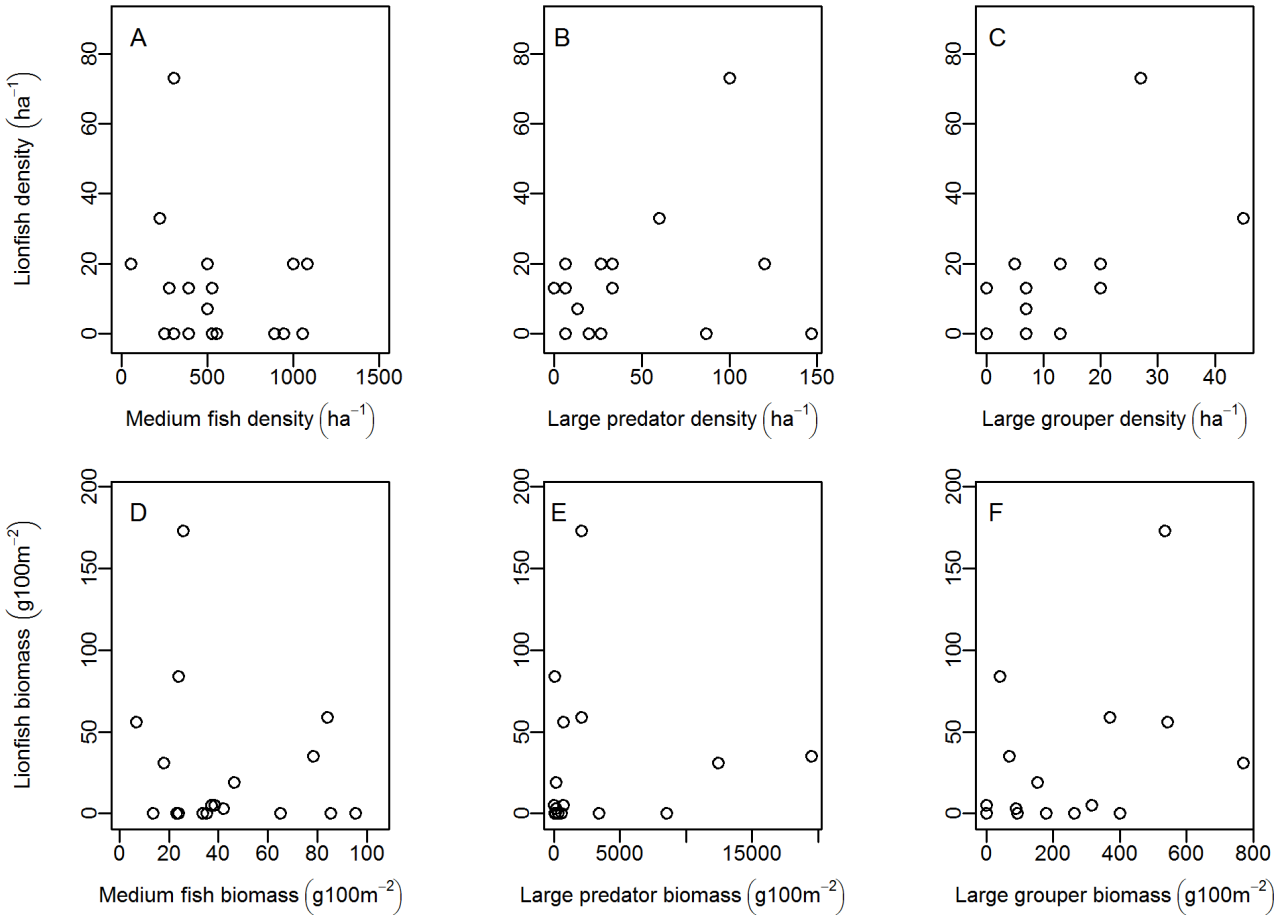
Effect of fish abundance (density or biomass) on lionfish abundance (density or biomass). Relationships between lionfish density and (A) medium fish density (individuals ha^−1^), (B) large predator density (individuals ha^−1^), and (C) large grouper density (individuals ha^−1^). Also relationships between lionfish biomass (g 100 m^−2^) and (D) medium fish biomass (g 100 m^−2^), (E) large predator biomass (g 100 m^−2^), and (F) large grouper biomass (g 100 m^−2^).

**Figure 4:**
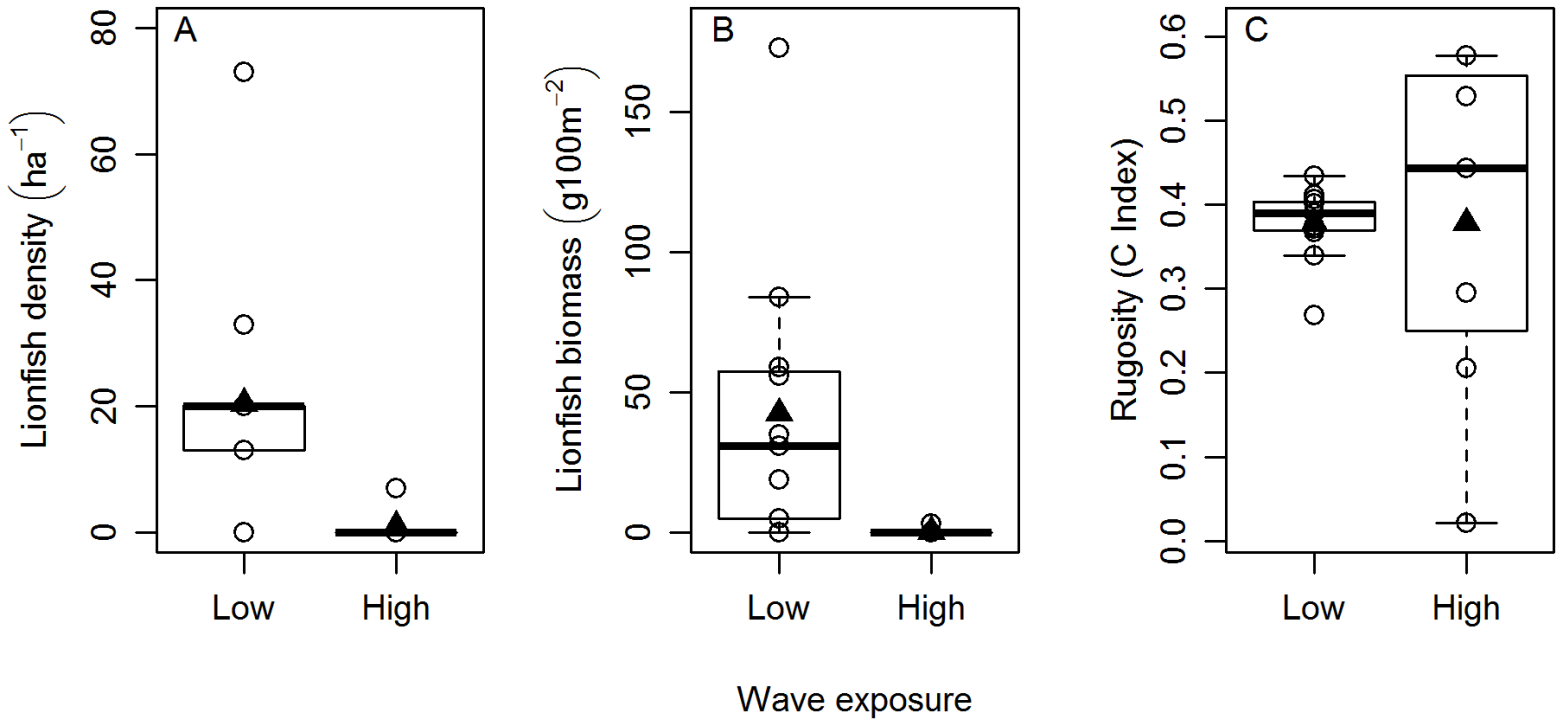
Effect of wave exposure (low/high) on lionfish abundance (density or biomass) and rugosity. Box plot of the relationships between wave exposure (categorized as low or high) and (A) lionfish density (individuals ha^−1^), (B) lionfish biomass (g 100 m^−2^), and (C) reef rugosity (C Index). Triangles denote mean values and open circles represent the mean value on each study site.

Our statistical models relating lionfish abundance across sites to abundances of various groupings of fishes and two environmental predictors help uncover possible functional relationships affecting lionfish invasion success. Lionfish density was negatively related to wave exposure, positively related to large predator abundance and did not exhibit any response to reef rugosity, medium fish- or large grouper- density (Table 1, Fig. 3, and Fig. 4). Lionfish biomass was also negatively related to wave exposure (Table 1 and Fig. 4) and did not exhibit any response to reef rugosity or the biomass of any of the fish groups (medium fish-, large predatory fish-, and large grouper- biomass; Table 1 and Fig. 3).

Correlations of the environmental predictors (rugosity and wave exposure) and benthic habitat type (the % cover of corals, the *Orbicella* habitat, macroalgae, turf algae, sponges and gorgonias) helped to identify direct effects of the environment on the benthos and the associated, potential indirect effects on lionfish invasion success. Only two benthic habitat types were correlated to one environmental predictor: macroalgal cover was negatively correlated to wave exposure in contrast with turf algae that was positively correlated to wave exposure (Table 3).

Additional analyses performed after segregating wave exposure into two categories, low and high, confirmed the results obtained in our previous statistical analysis, where wave exposure was included as a continuous predictor. Lionfish abundance (density and biomass) differed significantly between low and high wave-exposure environments (z = −2.906, p = 0.0036, df = 4 and z = −3.141, p = 0.0016, df = 4 for lionfish density and biomass respectively; Fig. 4). Average lionfish density was more than 22 times higher on low than on high wave exposure sites (20.6 versus 0.93 ha^−1^) (Fig. 4). Lionfish biomass exhibited an even more extreme pattern of a 122-fold higher average level in high versus low wave exposure environments (44.45 versus 0.36 g 100 m^−2^; Fig. 4), a consequence of finding larger lionfish at the sheltered sites as compared with the high wave exposure sites. We did not detect differences in reef rugosity between low and high wave exposure environments (t = −0.0131, p = 0.5051, df = 16).

## Discussion

Environmental wave exposure had a large influence on lionfish density and biomass (Fig. 4). Sheltered sites had a 22-fold higher density and a more than 120-fold greater biomass of lionfish than the exposed sites (Fig. 4). The apparent inhibition of lionfish invasion success in the sites with the highest wave exposure may reflect direct impacts of physical stresses on the lionfish. Some other fishes are also scarce in wave-exposed environments, perhaps because the energetic costs of locomotion may be a considerable barrier to occupation [40], [41]. In addition, when lionfish are hunting, they hover over or near their potential prey, and usually flare and spread their oversized, interconnected pectoral fins before striking (*personal observation*). They often blow jets of water at their prey, presumably to disorient them before striking [42]. These complex and sophisticated hunting behaviors could be difficult under conditions of high water velocities or strong oscillatory water motion. Our results agree with a recent study [43] that reported that “wind exposure has a weak negative effect on lionfish abundance”. However, we found that wave exposure significantly suppressed lionfish abundance and biomass, indicating that lionfish invasion success can be strongly affected by physically energetic conditions. If confirmed by subsequent experiments that lionfish are sensitive to hydrodynamic perturbations, it would imply some optimism that energetic environments may serve as refuges for coral reef fish populations even as lionfish may fundamentally modify fish communities in more protected environments [18].

Our findings of low lionfish abundances in the sites with relatively high wave exposure can not be explained by more effective lionfish removals by divers. To our knowledge, no lionfish derbies, like the ones organized by the Reef Environmental Education Foundation (REEF), have been held in San Salvador. [23] reported lower abundances of lionfish inside marine reserves than in several control-fished sites in the Caribbean and attributed the low lionfish densities within the marine parks to targeted and regular culling of the invasive fish by managers, dive operators, and/or tourist. However, none of our study sites were within marine reserves or regularly visited by dive operations, fishermen, and/or tourist (local fishermen and divers, *personal communication*). Therefore, at least in this Bahamian island, we can not attribute the low lionfish densities found in the wave-exposed sites to sustained lionfish removals.

The rugosity of coral reefs can be shaped by wave exposure, where coral reefs in sheltered locations are usually more structurally complex than reefs on wave-exposed environments [44]–[47]. However, we found that the rugosity on the reefs around San Salvador island appeared to be unaffected by wave exposure (Fig. 4). Two non-mutually exclusive hypotheses might explain these results. First, the relationship between habitat complexity and wave exposure might have been undetected due to our marginal sample size (n = 3). Second, benthic structural complexity is affected by wave exposure but also by depth, which can have a positive effect on reef rugosity [48]. The depth of our sites in San Salvador ranged from 13 m to 17 m, which might have been sufficient to buffer the differences in reef rugosity between exposed and sheltered environments ([48], Fig. 4). In fact, while swimming and diving around some of our wave-exposed study sites we noticied that the reef structure was visibly flatter in shallower areas than at the depth range where we were performing our surveys. Nonetheless, reef rugosity was unrelated to lionfish density and biomass, implying that structural complexity does not seem to facilitate lionfish invasion success (Table 1 and Fig. 4). These results agree with a recent study [43] that shows no correlation between habitat structural complexity and lionfish density. Reef rugosity in the [43] study was determined by visually assessing a substratum complexity category [49], which provides a limited qualitative estimation of habitat complexity. Our measurements of topographic complexity using the “chain and tape” method represent a fine scale quantification of reef rugosity [50]–[52]. Hence, our observations in San Salvador support the hypothesis that structural complexity is not a proximate driver of lionfish abundance.

The apparent inhibition of lionfish at the sites with high wave exposure could also occur indirectly through environmentaly driven biotic variables. Macroalgae were more abundant on sites with low wave-exposure which was related with more small fish (Table 3). The greater abundance of macroalgae in reefs previously built by *Orbicella* (when it was living) in areas of low wave exposure has been previously shown [53]. If benthic habitat plays a significant role in affecting the success of lionfish with regard to wave exposure, it would also likely influence lionfish invasion success by indirectly providing refuge and prey for small fishes, which are themselves prey for lionfish [12]. Macroalgae may also provide invertebrate prey to lionfish directly and thus represent an alternative indirect effect facilitating greater lionfish invasion success on the protected sites of this Bahamian island. Small fish (<5 cm total length) density and biomass were negatively correlated to wave exposure (Table S3). Because wave exposure and small fish abundance were autocorrelated (Table S3), we were unable to examine the effect of small prey fish on lionfish invasion success. However, we found that lionfish abundance was not associated with medium fish abundance, which are also prey of lionfish, suggesting a potential lack of an indirect bottom-up control of lionfish invasion success on this island.

Medium fish abundance was included in our models as a predictor under the assumption that the medium fish community has not yet been affected by lionfish presence. We did not conduct prey fish data counts before the lionfish invasion of San Salvador and we can not discern if lionfish had already influenced their prey fish community. The mean lionfish density in our study was relatively low when compared to other locations in the Bahamas (mean±SD: 13±18 individuals ha^−1^ in our study in San Salvador versus 101±103 individuals ha^−1^ in New Providence Island [54]). In fact, our lionfish densities in San Salvador were comparable to just a few sites with the lowest abundance of lionfish in New Providence. Another study in New Providence Island [18] reported that the detrimental effect of lionfish on native fish communities in coral reefs can be pronounced and quick: a 65% decline in the biomass of lionfish's prey fishes was quantified six years after the first lionfish sighting on nine coral reefs. On these reefs in New Providence, lionfish reached an abundance of nearly 40% of the total predator biomass in the system [18]. However, lionfish biomass in our sites in San Salvador represented only 1% of the large predator abundance in the reefs (Table 2) and these lionfish abundances appear to have been fairly low since the lionfish invasion of the island in 2005 (*personal communication* with local divers). Hence, it is likely that the effect of predatory lionfish on the medium fish communities of San Salvador 4 years after their arrival was limited.

Lionfish density was positively related to density of large native predatory fishes, but lionfish biomass was not associated with either large predatory fish density or biomass. The relationship between lionfish abundance and abundance of large predatory fishes imples a limited impact of competition and perhaps also predation on lionfish invasion success on this island. Instead, this positive relationship may arise indirectly through joint influences of some other variable on both lionfish and large native predatory fishes. For instance, predatory fish are often more abundant in sheltered environments [40], [48], which also seems to be the case for invasive lionfish. It is interesting that this positive relationship between the abundance of lionfish and native large predatory fishes exists even if fishing effects on larger predators may be higher in sheltered environments (because of better accessesability for fishermen) than on wave-exposed habitats. Therefore, the quantified abundances of large predators in the sheltered sites might have been relatively low compared to historic densities.

The lack of an effect of grouper abundance (density or biomass), which included only those fish >30 cm in total length, on lionfish abundance suggests that on San Salvador native predatory groupers are not providing biotic resistance against lionfish invasion, as shown in [24]. The limited top-down effects on lionfish found in San Salvador may not be surprising given the potent venom that lionfish carry in their dorsal, anal, and pelvic spines [55]. Although the act of any predation on healthy, free-roaming lionfish has not yet been reported, numerous studies of another successful toxic invader, the cane toad invading Australia, show low predation in the newly established range [56], [57].

The lack of a negative relationship between grouper abundance and lionfish abundance in our study contrasts with the conclusions in [24] from their study of lionfish and grouper biomass at sites along a chain of the Exuma Cays, also in the Bahamas. The Exuma reef sites included two sets: one in the Exuma Cays Land and Sea Park (ECLSP), where native grouper biomass is now high after protection from fishing, and another set to the north, where fishing continues and grouper biomass is lower. The authors concluded that when protected from fishing for long enough to rebuild grouper population biomass, predation by these native groupers can suppress the proliferation of lionfish on Exuma reefs. Grouper biomass in the Exuma protected area was on average approximately 9 times what we documented in San Salvador, so our failure to detect suppression of lionfish proliferation on this island could be explained by the relatively low biomass of native groupers. In addition, a recent study in Little Cayman Island reports predation by two native predatory fish species, Nassau grouper (*Epinephelus striatus*) and nurse shark (*Ginglymostoma cirratum*), on tethered but healthy lionfish [58], suggesting that predation of lionfish in the Caribbean might already be occurring. While the question of whether Atlantic native fish predators might exert a top-down control on invasive lionfish deserves further empirical investigation, the restoration and preservation of potential lionfish predators, in combination with selected removals of this invader [59], [60], are useful conservation efforts to manage the lionfish invasion of the Caribbean. In addition, energetic environments might impose direct or indirect resistance to the lionfish invasion, serving as fundamental refuge for native coral reef fish populations.

## Supporting Information

Figure S1 **Diagram of fish field surveys.** Diagram depicting the area of the different quadrats used on the fish surveys performed in the field.
(DOCX)

Table S1 **Prey fish species of** ***Pterois volitans*** **in the Atlantic reported in the scientific literature.**
(DOCX)

Table S2 **List of the fish species include in each of the fish categories: small fish (** ***a*** **), medium fish (** ***b*** **), large predatory fish (** ***c*** **) and large grouper (** ***d*** **).** a and b are the allometric length-weight parameters used to convert fish lenth into biomass.
(DOCX)

Table S3 **Table of the Pearson's product-moment correlation coefficients between the biotic (density and biomass of small fish, medium fish, large predatory fish and large grouper) and environmental (wave exposure and rugosity) model predictors.** ***d*** **indicates density in individual 1500 m^−2^ and** ***b*** **indicates biomass in g 100 m^−2^.** The asterisk (*) indicates significant differences at p-values <0.05.
(DOCX)
