# Supplementary material for: Environmental and Biotic Correlates to Lionfish Invasion Success in Bahamian Coral Reefs
Source: PLoS One. 2014 Sep 3;9(9):e106229. doi: 10.1371/journal.pone.0106229 (PMC4153550; doi:10.1371/journal.pone.0106229)

Fig. S1. Diagram depicting the area of the different quadrats used on the fish counts performed in the field.


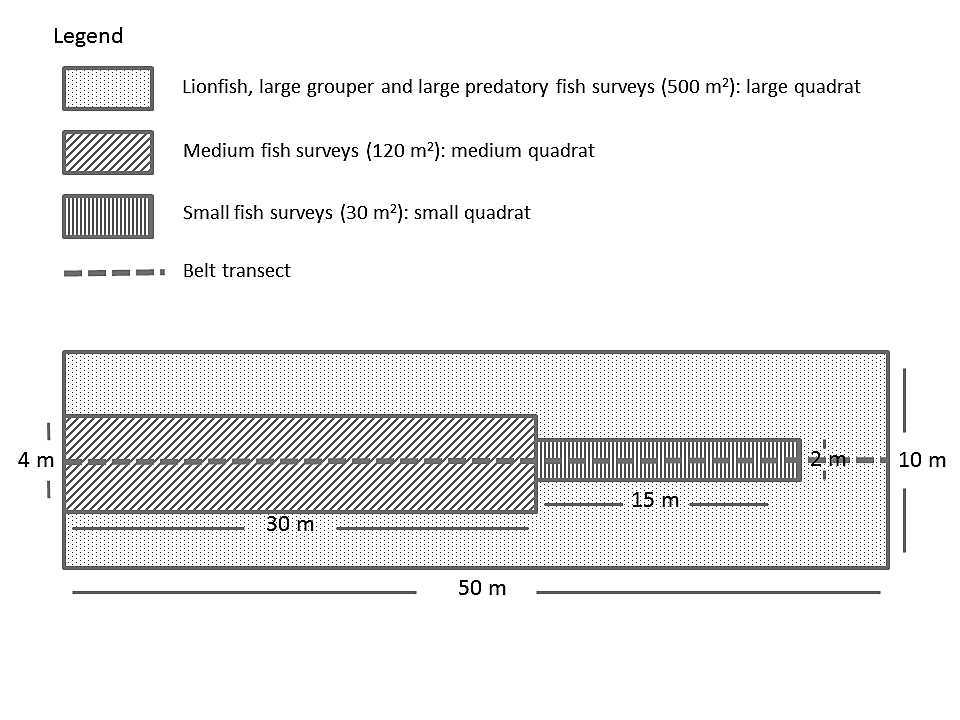

Supplement: Figure S1 — Diagram of fish field surveys. Diagram depicting the area of the different quadrats used on the fish surveys performed in the field. (DOCX) [file pone.0106229.s001.docx]
