# Supplementary material for: Environmental and Biotic Correlates to Lionfish Invasion Success in Bahamian Coral Reefs
Source: PLoS One. 2014 Sep 3;9(9):e106229. doi: 10.1371/journal.pone.0106229 (PMC4153550; doi:10.1371/journal.pone.0106229)
Supplement: Table S1 — Prey fish species of Pterois volitans in the Atlantic reported in the scientific literature. (DOCX) [file pone.0106229.s002.docx]

Table S1. Prey fish species of *Pterois volitans* in the Atlantic reported in the scientific literature.

| Prey fish family | Prey fish species | Reference |
| --- | --- | --- |
| Acanthuridae | *Acanthurus bahianus* | Morris and Akins 2009, Green et al. 2011 |
| Acanthuridae | *Acanthurus chirurgus* | Green et al. 2011, Green et al. 2011 |
| Acanthuridae | *Acanthurus coeruleus* | Green et al. 2011 |
| Apogonidae | *Apogon townsendi* | Morris and Akins 2009, Cote et al. 2013 |
| Apogonidae | *Apogon pseudomaculatus* | Muñoz et al. 2011 |
| Apogonidae | *Apogon binotatus* | Morris and Akins 2009, Green et al. 2011, Cote et al. 2013 |
| Aulostomidae | *Aulostomus maculatus* | Morris and Akins 2009, Cote et al. 2013 |
| Blenniidae | *Acanthemblemaria aspera* | Cote et al. 2013 |
| Blenniidae | *Lucayablennius zingaro* | Morris and Akins 2009 |
| Blenniidae | *Malacoctenus triangulatus* | Morris and Akins 2009 |
| Blenniidae | *Hypleurochilus geminates* | Muñoz et al. 2011 |
| Blenniidae | *Labrisomus haitiensis* | Cote et al. 2013 |
| Blenniidae | *Malacoctenus boehlkei* | Morris and Akins 2009, Cote et al. 2013 |
| Blenniidae | *Malacoctenus gilli* | Green et al. 2011 |
| Bothidae | *Bothus ocellatus* | Cote et al. 2013 |
| Carangidae | *Selar crumenophthalmus* | Muñoz et al. 2011 |
| Chaetodontidae | *Chaetodon capistratus* | Green et al. 2011 |
| Cirrhitidae | *Amblycirrhitus pinos* | Morris and Akins 2009 |
| Gobiidae | *Coryphopterus personatus* | Morris and Akins 2009, Cote et al. 2013 |
| Gobiidae | *Coryphopterus bol* | Cote et al. 2013 |
| Gobiidae | *Coryphopterus eidolon* | Morris and Akins 2009, Cote et al. 2013 |
| Gobiidae | *Coryphopterus dicrus* | Morris and Akins 2009 |
| Gobiidae | *Coryphopterus glaucofraenum* | Albins and Hixon 2008, Morris and Akins 2009, Cote et al. 2013 |
| Gobiidae | *Lythrypnus spilus* | Cote et al. 2013 |
| Gobiidae | *Priolepis hipoliti* | Morris and Akins 2009, Green et al. 2011, Cote et al. 2013 |
| Gobiidae | *Gnatholepis thompsoni* | Albins and Hixon 2008, Green et al. 2011, Cote et al. 2013 |
| Grammatidae | *Gramma loreto* | Morris and Akins 2009, Green et al. 2011, Cote et al. 2013 |
| Grammatidae | *Gramma melacara* | Morris and Akins 2009 |
| Haemulidae | *Haemulon aurolineatum* | Green et al. 2011, Muñoz et al. 2011 |
| Haemulidae | *Haemulon flavolineatum* | Green et al. 2011 |
| Haemulidae | *Haemulon plumierii* | Green et al. 2011, Anton 2013 |
| Haemulidae | *Haemulon melanurum* | Albins and Hixon 2008 |
| Holocentridae | *Holocentrus rufus* | Cote et al. 2013 |
| Holocentridae | *Sargocentron coruscum* | Cote et al. 2013 |
| Holocentridae | *Sargocentron vexillarium* | Morris and Akins 2009 |
| Inermiidae | *Inermia vittata* | Cote et al. 2013 |
| Labridae | *Thalassoma bifasciatum* | Morris and Akins 2009, Green et al. 2011, Anton 2013, Cote et al. 2013 |
| Labridae | *Halichoeres pictus* | Albins and Hixon 2008, Morris and Akins 2009 |
| Labridae | *Halichoeres bivittatus* | Morris and Akins 2009, Green et al. 2011, Cote et al. 2013 |
| Labridae | *Clepticus parrae* | Morris and Akins 2009, Cote et al. 2013 |
| Labridae | *Halichoeres garnoti* | Morris and Akins 2009, Green et al. 2011, Anton 2013, Cote et al. 2013 |
| Labridae | *Halichoeres maculipinna* | Morris and Akins 2009, Cote et al. 2013 |
| Labridae | *Bodianus rufus* | Morris and Akins 2009 |
| Labridae | *Xyrichtys* sp*.* | Morris and Akins 2009, Green et al. 2011 |
| Lutjanidae | *Ocyurus chrysurus* | Morris and Akins 2009 |
| Monacanthidae | *Monacanthus tuckeri* | Morris and Akins 2009, Cote et al. 2013 |
| Monacanthidae | *Monacanthus ciliatus* | Muñoz et al. 2011 |
| Mullidae | *Pseudupeneus maculatus* | Morris and Akins 2009 |
| Opistognathidae | *Opistognathus aurifrons* | Albins and Hixon 2008 |
| Pomacentridae | *Chromis insolata* | Morris and Akins 2009 |
| Pomacentridae | *Chromis cyanea* | Morris and Akins 2009, Cote et al. 2013 |
| Pomacentridae | *Chromis multilineata* | Morris and Akins 2009, Cote et al. 2013 |
| Pomacentridae | *Chromis enchrysura* | Muñoz et al. 2011 |
| Pomacentridae | *Stegastes partitus* | Morris and Akins 2009, Green et al. 2011, Cote et al. 2013 |
| Pomacentridae | *Stegastes leucostictus* | Albins and Hixon 2008, Morris and Akins 2009, Green et al. 2011 |
| Pomacentridae | *Stegastes variabilis* | Morris and Akins 2009, Cote et al. 2013 |
| Pomacentridae | *Stegastes diencaeus* | Green et al. 2011 |
| Scaridae | *Scarus iserti* | Morris and Akins 2009, Green et al. 2011 |
| Scaridae | *Sparisoma radians* | Muñoz et al. 2011 |
| Scaridae | *Sparisoma viride* | Morris and Akins 2009, Green et al. 2011 |
| Scaridae | *Sparisoma aurofrenatum* | Green et al. 2011, Cote et al. 2013 |
| Scorpaenidae | *Pterois volitans* | Cote et al. 2013 |
| Serranidae | *Cephalopholis cruentata* | Cote et al. 2013 |
| Serranidae | *Epinephelus striatus* | Morris and Akins 2009 |
| Serranidae | *Serranus tigrinus* | Morris and Akins 2009, Muñoz et al. 2011, Cote et al. 2013 |
| Serranidae | *Hypoplectrus* sp*.* | Morris and Akins 2009 |
| Serranidae | *Liopropoma rubre* | Morris and Akins 2009, Cote et al. 2013 |
| Serranidae | *Serranus phoebe* | Muñoz et al. 2011 |
| Serranidae | *Serranus subligarius* | Muñoz et al. 2011 |
| Serranidae | *Diplectrum* sp*.* | Muñoz et al. 2011 |
| Serranidae | *Serranus baldwini* | Muñoz et al. 2011 |
| Serranidae | *Schultzea beta* | Muñoz et al. 2011 |
| Synodontidae | *Synodus synodus* | Cote et al. 2013 |
| Synodontidae | *Saurida normani* | Muñoz et al. 2011 |
| Tetradontidae | *Canthigaster rostrata* | Morris and Akins 2009, Green et al. 2011 |
| Tripterygidae | *Enneanectes* sp*.* | Morris and Akins 2009 |
