# Supplementary material for: Environmental and Biotic Correlates to Lionfish Invasion Success in Bahamian Coral Reefs
Source: PLoS One. 2014 Sep 3;9(9):e106229. doi: 10.1371/journal.pone.0106229 (PMC4153550; doi:10.1371/journal.pone.0106229)
Supplement: Table S2 — List of the fish species include in each of the fish categories: small fish ( a ), medium fish ( b ), large predatory fish ( c ) and large grouper ( d ). a and b are the allometric length-weight parameters used to convert fish lenth into biomass. (DOCX) [file pone.0106229.s003.docx]

Table S2. List of the fish species include in each of the fish categories: small fish (*a*), medium fish (*b*), large predatory fish (*c*) and large grouper (*d*). a and b are the allometric length-weight parameters used to convert fish lenth into biomass.

| Fish species scientific name | a | b |
| --- | --- | --- |
| *Acanthurus bahianus^a,b^* | 0.025 | 2.9 |
| *Acanthurus chirurgus^b^* | 0.0204 | 2.92 |
| *Acanthurus coeruleus^a,b^* | 0.032 | 2.95 |
| *Aulostomus maculatus^b,c^* | 0.0039 | 2.866 |
| *Bodianus rufus^b^* | 0.014 | 3.053 |
| *Canthigaster rostrata^b^* | 0.0513 | 2.72 |
| *Caranx latus^c^* | 0.0417 | 2.79 |
| *Caranx lugubris^c^* | 0.024 | 2.91 |
| *Carcharhinus limbatus^c^* | 0.0087 | 2.96 |
| *Carcharhinus perezii^c^* | 0.0271 | 3 |
| *Cephalopholis cruentata^b,c^* | 0.011 | 3.11 |
| *Chaetodon capistratus^b^* | 0.023 | 3.19 |
| *Chromis cyanea^a,b^* | 0.018 | 3 |
| *Chromis multilineata^a,b^* | 0.018 | 3 |
| *Epinephelus striatus^b,c,d^* | 0.0091 | 3.16 |
| *Gramma loreto^a^* | 0.0011 | 3.04 |
| *Haemulon flavolineatum^b^* | 0.018 | 2.99 |
| *Haemulon plumierii^b^* | 0.0204 | 2.95 |
| *Halichoeres bivittatus^a,b^* | 0.0112 | 3.05 |
| *Halichoeres garnoti^a,b^* | 0.0052 | 3.375 |
| *Halichoeres maculipinna^b^* | 0.0027 | 3.693 |
| *Kyphosus secatator^c^* | 0.0174 | 3.079 |
| *Lutjanus analis^c^* | 0.0151 | 3.02 |
| *Lutjanus apodus^c^* | 0.0182 | 3 |
| *Mycteroperca tigris^c,d^* | 0.0135 | 3.12 |
| *Pseudupeneus maculatus^b^* | 0.0158 | 3.05 |
| *Sargocentron coroscum^b^* | 0.0141 | 3.04 |
| *Scarus iserti^b^* | 0.058 | 3.02 |
| *Seriola rivoliana^c^* | 0.018 | 2.94 |
| *Serranus tigrinus^a^* | 0.0145 | 3.048 |
| *Sparisoma aurofrenatum^a,b^* | 0.0117 | 3.15 |
| *Sparisoma viride^a,b^* | 0.0257 | 2.93 |
| *Sphyraena barracuda^c^* | 0.0095 | 2.95 |
| *Stegastes diencaeus^a,b^* | 0.024 | 2.98 |
| *Stegastes leucostictus^a,b^* | 0.0288 | 2.97 |
| *Stegastes partitus^a,b^* | 0.0182 | 3.152 |
| *Thalassoma bifasciatum^a,b^* | 0.011 | 2.97 |
