# Supplementary material for: Environmental and Biotic Correlates to Lionfish Invasion Success in Bahamian Coral Reefs
Source: PLoS One. 2014 Sep 3;9(9):e106229. doi: 10.1371/journal.pone.0106229 (PMC4153550; doi:10.1371/journal.pone.0106229)
Supplement: Table S3 — Table of the Pearson's product-moment correlation coefficients between the biotic (density and biomass of small fish, medium fish, large predatory fish and large grouper) and environmental (wave exposure and rugosity) model predictors. d indicates density in individual 1500 m−2 and b indicates biomass in g 100 m−2. The asterisk (*) indicates significant differences at p-values <0.05. (DOCX) [file pone.0106229.s004.docx]

Table S3. Table of the Pearson’s product-moment correlation coefficients between the biotic (density and biomass of small fish, medium fish, large predatory fish and large grouper) and abiotic (wave exposure and rugosity) model predictors. *d* indicates density in individual 1500 m^-2^ and *b* indicates biomass in g 100 m^-2^. The asterisk (*) indicates significant differences at p-values <0.05.

|  | Small fish d | Medium fish d | Large predator d | Large grouper d | Rugosity | Wave exposure |
| --- | --- | --- | --- | --- | --- | --- |
| Small fish d |  | -0.005 | -0.058 | 0.047 | 0.052 | *-0.507 |
| Medium fish d |  |  | -0.151 | -0.339 | -0.141 | 0.127 |
| Large predator d |  |  |  | *0.604 | 0.232 | -0.049 |
| Large grouper d |  |  |  |  | -0.016 | -0.244 |
| Rugosity |  |  |  |  |  | -0.03 |
| Wave exposure |  |  |  |  |  |  |
|  | Small fish b | Medium fish b | Large predator b | Large grouper b | Rugosity | Wave exposure |
| Small fish b |  | 0.129 | -0.029 | -0.071 | 0.037 | *-0.546 |
| Medium fish b |  |  | 0.054 | -0.399 | -0.108 | 0.063 |
| Large predator b |  |  |  | 0.2483 | 0.095 | -0.34 |
| Large grouper b |  |  |  |  | 0.033 | -0.135 |
| Rugosity |  |  |  |  |  | -0.03 |
| Wave exposure |  |  |  |  |  |  |
